# A Mobile Sexual Health App on Empowerment, Education, and Prevention for Young Adult Men (MyPEEPS Mobile): Acceptability and Usability Evaluation

**DOI:** 10.2196/17901

**Published:** 2020-04-07

**Authors:** Brittany Gannon, Rindcy Davis, Lisa M Kuhns, Rafael Garibay Rodriguez, Robert Garofalo, Rebecca Schnall

**Affiliations:** 1 School of Nursing Columbia University New York, NY United States; 2 HIV Center for Clinical and Behavioral Studies New York State Psychiatric Institute New York City, NY United States; 3 Gertrude H Sergievsky Center Columbia University New York, NY United States; 4 Lurie Children's Hospital Chicago, IL United States; 5 Ann & Robert H Lurie Children’s Hospital of Chicago Northwestern University Feinberg School of Medicine Chicago, IN United States

**Keywords:** young adults, usability, HIV, mHealth, young men, mobile phone

## Abstract

**Background:**

HIV incidence among young adult men who have sex with men (MSM), particularly among black and Latino men, continues to rise. As such, continued HIV prevention interventions for young MSM of color are of utmost importance. Male Youth Pursuing Empowerment, Education and Prevention around Sexuality (MyPEEPS) Mobile is a comprehensive HIV prevention and sexual health education smartphone app initially created to promote sexual health and HIV prevention among adolescent sexual minority young men aged 13 to 18 years.

**Objective:**

The objective of this study was to critically appraise the acceptability and usability of MyPEEPS Mobile for young adult MSM aged 19 to 25 years.

**Methods:**

Study participants used the mobile app, completed usability questionnaires and in-depth interviews, and reported their experience using the app. Analysis of interview data was guided by the Unified Theory of Acceptance and Use of Technology (UTAUT) to better understand the usability and acceptability of this intervention for young adults. Interview data were coded using the following constructs from the UTAUT model: performance expectancy, effort expectancy, and social influence.

**Results:**

A total of 20 young adult MSM (n=10 in Chicago, Illinois, and n=10 in New York, New York) were enrolled in the study. Participants reported that MyPEEPS Mobile was free of functional problems (Health Information Technology Usability Evaluation Scale scores and Post-Study System Usability Questionnaire scores consistent with high usability), easy to use, and useful, with an engaging approach that increased acceptability, including the use of avatars and animation, and inclusive representation of the diverse identities by race and ethnicity, gender identity, and sexual orientation. Recommended areas for improving MyPEEPS Mobile for the target demographic included more adult-oriented graphics, advanced educational content, scenarios for youth with more sexual experience, and search function to increase accessibility of key content.

**Conclusions:**

Overall, young adult MSM aged 19 to 25 years described the MyPEEPS Mobile as educational, informative, and usable for their sexual health education and HIV prevention needs, and they provided actionable recommendations to optimize its use and applicability for this age group.

## Introduction

### Background

Of the 38,739 new HIV diagnoses in the United States and dependent areas, young men, aged 13 to 24 years, accounted for 18% of new diagnoses of HIV in 2017 [1]. Despite continued advances in HIV prevention due, in part, to the widespread promotion of treatment as prevention [2,3] for HIV-positive individuals and pre-exposure prophylaxis (PrEP) [4,5] for high-risk HIV-negative individuals, young men who have sex with men (YMSM) continue to bear the burden of new HIV infections. Of the young men diagnosed with HIV in 2017, 21% were aged 15 to 19 years and 79% were aged 20 to 24 years, with 93% of male cases attributed to male-to-male sexual contact [6]. Further highlighting the severity of the epidemic among subpopulations, young black/African American and Hispanic/Latino youth account for the highest proportion of new HIV diagnoses [6]. Given the racial/ethnic disparities in the epidemic, access to culturally and developmentally appropriate HIV prevention tools for YMSM of color is vital to *Ending the HIV Epidemic: A Plan for America* initiative in the United States, as proposed by the Department of Health and Human Services [7].

Sexual risk factors among YMSM identified in the literature include low rates of HIV testing; high rates of sexually transmitted infections (STIs), particularly among youth of color aged 20 to 24 years; substance use; as well as low levels of consistent condom and PrEP use [6,8]. YMSM face additional multilevel challenges to accessing effective sexual health education and services, including low risk awareness, intersectional stigma, poor access to care, high cost of care, medical mistrust, homelessness, and lack of culturally appropriate care [9-12].

According to the US 2020 National HIV/AIDS Strategy, public health initiatives should focus on increasing HIV and STI testing, linkage to care, universal viral suppression, and increasing PrEP awareness and access [13]. In addition to these strategies, access to HIV prevention information via Web and mobile platforms has been shown to improve knowledge, acceptability, and utilization of health prevention interventions and services [14].

In consideration of the 2020 National HIV/AIDS Strategy, several culturally tailored mobile health (mHealth) technology interventions have been designed in areas of primary HIV prevention (eg, myDEx, *Male Youth Pursuing Empowerment, Education and Prevention around Sexuality* [MyPEEPS], and HealthMindr), HIV testing and PrEP uptake (eg, P3: Prepared, Protected, emPowered; Mychoices; and mLab), and *linkage to care* (eg, LYNX and Get Connected) and are in various stages of testing for feasibility, acceptability, and efficacy [14-20]. Advantages of mHealth apps include the ability to rapidly and cost-effectively disseminate information broadly while also addressing the stated needs and desires of men who have sex with men (MSM) [14,21,22]. Moreover, use of mobile apps for sexual health education may facilitate privacy for YMSM while accessing sensitive and often stigmatized health information [23].

### Objective

The *MyPEEPS* curriculum was originally developed as an in-person group-based intervention for racially and ethnically diverse YMSM to improve HIV-related risk [24]. For the group-based intervention, MyPEEPS was manualized and consisted of six interactive sessions focusing on HIV and STI epidemiology in YMSM, building knowledge and skills for safer sex, minority stress, emotion regulation, interpersonal and substance-related risk factors, developing risk reduction plans, and condom negotiation. MyPEEPS Mobile was adapted from the in-person, group-based intervention to a mobile app via an iterative process, including expert panel reviews, in-depth interviews with adolescent YMSM aged 13 to 18 years [25], a rigorous usability evaluation [26], and pilot testing [18].

MyPEEPS Mobile is a mobile, responsive Website that is viewable on small screens and usable with touch screens. MyPEEPS Mobile provides educational information about STIs and HIV for YMSM, builds skills for condom use, and raises awareness of minority stress. The app content is guided by 4 peeps: Tommy, Philip, Nico, and Artemio, or composite characters/avatars, who relay content through comics, animation, and scenarios delivered through 21 brief activities in four sequential modules (see Multimedia Appendix 1). A running theme throughout the intervention is the “Bottom Line,” in which participants can set goals about their sexual risk reduction and commit to how much sexual risk they are willing to undertake. Privacy is protected via password and automatic log-off of the app after 20 min of inactivity.

In this study, we sought to assess the usability of MyPEEPS Mobile among an older age group with increased risk for HIV infection. To do so, we conducted in-depth interviews about the app content with young adult MSM aged 19 to 25 years.

## Methods

### Study Period and Participant Inclusion

This study was approved by the Institutional Review Board (IRB) at Columbia University Medical Center, which served as the single IRB of record. Data were collected in New York, New York, from August 2018 to October 2018 and in Chicago, Illinois, from December 2018 to February 2019. An a priori sample size of approximately 20 was estimated to provide saturation of acceptability and usability themes [27]. Individuals were recruited for participation via advertisement on social media platforms (ie, Instagram, Grindr, etc), via flyer distribution at local community locations and events (New York City and Chicago), and among research participants from other studies at the two sites. Inclusion criteria were as follows: (1) aged 19 to 25 years, (2) male sex assigned at birth, (3) self-identified as male or gender nonconforming/nonbinary, (4) comfortable speaking and reading in English, (5) living within the metropolitan area of New York City or Chicago, (6) anal or oral sex with another male in the past 12 months, and (7) self-reported HIV-negative or unknown status. Interested individuals were screened for participation via a Web or phone screening survey and, if eligible, enrolled in a subsequent in-person study visit.

### Data Collection

Participants met the study staff at each study site for a single visit, lasting approximately 3 to 4 hours. The interviews were conducted in private conference rooms at the Lurie Children’s Hospital and the Columbia University School of Nursing. After initial informed consent and completion of a computer-assisted self-interview, including demographic and behavioral questions, participants were instructed to complete intervention activities in the MyPEEPS Mobile app. While using MyPEEPS Mobile, participants recorded notes on their perceptions of app design and content. After completing the modules, participants completed the following usability questionnaires: (1) Health Information Technology Usability Evaluation Scale (Health-ITUES) [28] and (2) Post-Study System Usability Questionnaire (PSSUQ) [29,30]. Participants then completed face-to-face, audio-recorded, qualitative interviews, facilitated by study staff, using a semistructured interview guide. The following questions were included in the guide: (1) Thinking back about the information you learned from the MyPEEPS app, how would you apply this information/lessons/activities in your own life?; (2) How do the MyPEEPS activities reflect your cultural beliefs, norms, values?; (3) How do you perceive this app would be of relevance to other young adult MSM aged 19 to 25 years?; and (4) How would you modify these activities, if needed, to make them more relevant to young adult MSM aged 19 to 25 years? Data were collected until saturation was reached.

### Theoretical Model

The Unified Theory of Acceptance and Use of Technology (UTAUT) model was originally developed as a conceptual framework to explain individuals’ intention to adopt and use technological innovations. In this study, we draw from this theory to describe the applicability and likelihood of use of the MyPEEPS Mobile app among young adult MSM aged 19 to 25 years [31-33]. Originally developed to explain employee adoption of technology in the workplace, the UTAUT model has been extended to new contexts, such as health information systems and new populations (eg, consumers and health care professionals) [34,35]. Here, we use the UTAUT model to evaluate the applicability of MyPEEPS for young adult MSM in community settings where they access and use mobile apps. The following key theoretical constructs from the UTAUT model guided the analysis of the in-depth interview data: (1) performance expectancy, (2) effort expectancy, and (3) social influence [32,33].

Performance expectancy is defined as the degree to which an individual believes that using the technology will help them attain gains in the outcome of interest, that is, work performance in the original conceptualization, and herein, health protective behavior. Effort expectancy is the degree of ease associated with use of the technology. Finally, social influence is the extent to which an individual perceives that important others believe they should use the technology. These constructs are theorized to drive behavioral intention, which leads to use behavior. That is, if users expect use of the technology to improve the outcome of interest, find it easy to use, and perceive that important others believe they should use it, use of the technology will follow. Several constructs that stem from organizational contexts used in the original model (ie, facilitating conditions and extrinsic motivation) were eliminated in this study, as they were not applicable because use of the app does not depend on an organizational structure to drive use.

In addition to the primary constructs, the UTAUT model also includes moderators that are theorized to impact the pathway from primary constructs’ technology use, based on individual characteristics. For this study, we sought to identify potential contextual factors that might also impact the pathway to use, based on analysis of interview data.

### Qualitative Analyses

The qualitative analysis process consisted of two rounds of coding, including primary, open coding of emergent themes and secondary, thematic coding of those themes onto UTAUT constructs [36]. Audio recordings were transcribed verbatim and coded by 2 independent coders (BG and RD) in NVivo qualitative data analysis software (QSR International Pty Ltd, version 12, 2018). Before coding, both reviewers wrote a reflexivity statement of their background, preconceived notions, and/or engagement in HIV research and lesbian, gay, bisexual, transgender, queer affairs. This form of self-appraisal is utilized to ensure rigor of the analyses and brings awareness to the interpretive lens through which a qualitative analysis is being performed [37].

**Figure 1:**
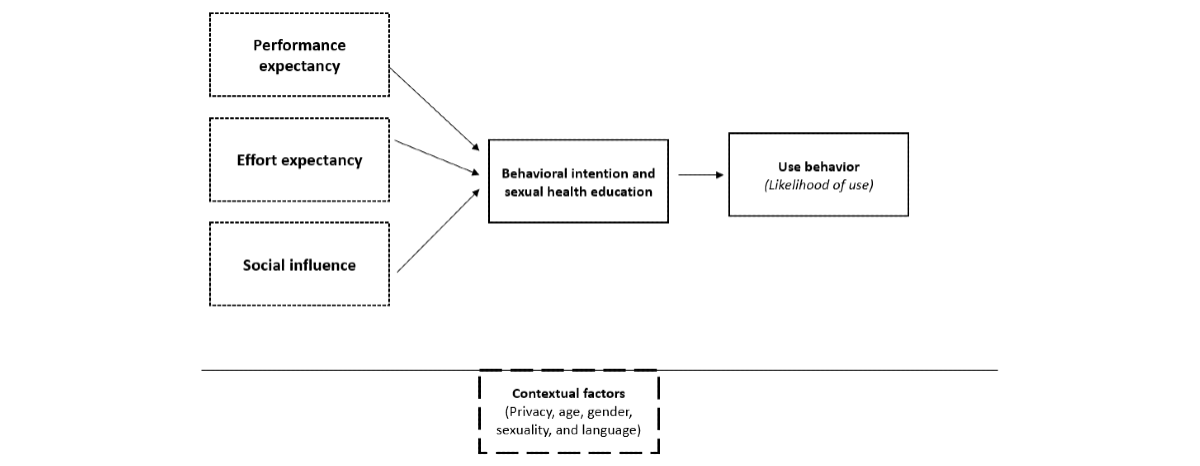
Adapted Unified Theory of Acceptance and Use of Technology model.

Initial content analyses via line-by-line coding, a form of open coding, was performed by the 2 coders independently to identify emergent codes [36]. The coders then met to debrief and discuss emergent codes derived from this process [38]. Secondary content analyses were then performed by applying the UTAUT as a semistructured framework; see Figure 1 [32,39]. Individual coders were tasked to apply themes within the UTAUT model—incorporating main constructs and subconstructs.

The coders met after completion of secondary analyses to discuss the fit of their codes and any variances in the application to the UTAUT model. Cognitive mapping of secondary codes (development of a graphic map that represents the relationships between concepts) was then performed in NVivo to facilitate discussion, integration, and agreement for the development of a preliminary version of the adapted model. Moreover, 3 of the 4 macro-constructs were agreed upon and utilized in the adapted model to explain the applicability and likelihood of use of MyPEEPS among young adult MSM, including (1) performance expectancy, (2) effort expectancy, and (3) social influence. Several constructs of the UTAUT model (ie, facilitating conditions, extrinsic motivation, etc) were not apparent in the interview data, and thus, they were not applied in the adapted model. To facilitate credibility and reliability, the study team, comprising the principal investigator, primary coders, and the study coordinator, then convened for consensus coding of UTAUT-related content. Applicability of constructs and codes were discussed with any discrepancies among codes negotiated and reached by majority [40,41]. An adapted version of the UTAUT model is presented in Figure 1, highlighting the thematic structure of the end user’s experience with MyPEEPS.

## Results

### Study Sample

The study sample comprised 20 YMSM, aged 19 to 25 years, recruited from New York (n=10) and Chicago (n=10), with a median age of 22.5 years. Regarding sexual orientation, 90% (18/20) identified as gay, 5% (1/20) identified as bisexual, and 5% (1/20) identified as pansexual. In terms of race/ethnicity, 50% (10/20) identified as white, 35% (7/20) black/African American, 10% (2/20) multiracial, and 5% (1/20) other (Puerto Rican). Of these participants, 45% (9/20) had completed college, 25% (5/20) had completed some college, 20% (4/20) had completed high school, 5% (1/20) was currently enrolled in high school, and 5% (1/20) had completed a master’s degree.

### Usability Ratings

Overall, participants rated the app as highly usable on the usability instruments. On the PSSUQ, all subscales, including system quality, information quality, and interface quality, fell below 2 on average, indicating high usability (see Table 1). The overall mean (SD) PSSUQ score on all 16 items was 1.83 (0.43). The subconstructs of the Health-ITUES, including system impact, perceived usefulness, perceived ease of use, and user control, were all rated above 4 on average, indicating that participants rated the app as highly usable (see Table 2). The mean (SD) Health-ITUES score on all 20 items was 4.41 (0.23).

**Table 1:** Post-Study System Usability Questionnaire scores (N=20).

PSSUQ^a^ constructs	Score^b^, mean (SD)
System quality	1.49 (0.15)
Information quality	1.98 (0.42)
Interface quality	1.48 (0.10)

^a^PSSUQ: Post-Study System Usability Questionnaire.

^b^Scores range from 1=strongly agree to 7=strongly disagree.

**Table 2:** Health Information Technology Usability Evaluation Scale scores.

Health-ITUES^a^ constructs	Score^b^, mean (SD)
System impact	4.37 (0.24)
Perceived usefulness	4.29 (0.16)
Perceived ease of use	4.67 (0.08)
User control	4.38 (0.28)

^a^Health-ITUES: Health Information Technology Usability Evaluation Scale.

^b^Scores range from 1=strongly disagree to 5=strongly agree.

### Qualitative Findings

Qualitative findings are arranged by the constructs of the UTAUT model. Quotes from study participants are presented below followed by their city of residence and their age in years.

### Performance Expectancy

Participants described their intention to use the app within their everyday lives. In particular, one participant said:

I think it’s applicable to anyone’s life who is sexually active. Obviously, the messaging was catered to a gay demographic. So, identifying as a gay male, I did find it applicable to my life; more specifically, my sex life. So, yeah, I did think that the information that was shared is something that I can use in my everyday life.

Chicago, 24 years

Performance expectancy and plans to use the MyPEEPS app were further operationalized through the three subconstructs of the UTAUT model: (1) perceived usefulness, (2) outcome expectations, and (3) relative advantage [32].

*Perceived usefulness* of MyPEEPS was ascertained from end users’ summative experiences and perceived knowledge attained while using the app. Most participants noted that the app was informative and useful beyond standard sexual health education, with statements such as:

I feel like even I learned something new today, so we could all learn something new.

New York, 21 years

Participants expressed a foreseeable usefulness of MyPEEPS for YMSM who live in different geographic regions (ie, outside of urban settings):

Obviously, in more conservative areas you still have like very strong conservative values on that. Like, the portion of sex ed that dealt with gay sex, was like five minutes, you know. So, I think it would be applicable for people who don’t have the same type of sex education because there’s still plenty of places that their sex education is still abstinence first, which is just not reality, you know.

New York, 19 years

On the other hand, participants who had more educational or life experience perceived the app as less useful. For example, one user noted:

For me it felt redundant because it didn’t change mine [bottom line] at all, but maybe for someone who is younger or has less sexual experience or education it could still...it could be helpful for them.

Chicago, 24 years

Another participant commented that the app was less useful to him because of his educational background:

Coming from a student background, especially in a school of public health, this is an activity that we had done before, so it was kind of a little bit repetitive for me just because I have seen these questions before, and I know what we’re doing here.

New York, 23 years

When probed by the interviewer regarding usefulness of the app, one end user commented:

So, I guess it’s important to clarify – what is the purpose of the app? Is it to be a one-time resource, or is it to be a go-back-to-it resource?

Chicago, 25 years

*Outcome expectations* were expectations regarding the impact of the use of the app on health behavior. After participants used the app, one user reflected his intent to change behavior with his partner:

Me and my lover have to plan. I know I will be looking at the app. When I get home, I am going to tell him all about it.

New York, 19 years

Furthermore, use of culturally representative avatars and animation surpassed participants’ expectations relative to expected traditional sexual health didactic content. One participant stated:

I really like “Testing with Tommy” where it literally...you go through what it’s like to go to an HIV clinic and go through that experience. Like, you take a number, and there are a bunch of people there. It’s like it’s not a big deal.

Chicago, 25 years

One of the major expectations “not met” by participants was the availability and accessibility of sexual health resources. A representative comment about this was as follows:

I really wish that all the references and everything could have been provided somewhere versus going back inside the modules.

New York, 23 years

Although sexual health–related external references and hotlines were provided in activities throughout the app, this did not meet end user’s expectation of having a single “go-to” resource tool, which was a content recommendation.

*Relative advantage* is the extent to which using the technology is perceived as better than using its precursor [32]. In this study, relative advantage included participants’ perceptions of the use of MyPEEPS relative to the current standards of sexual health education (ie, provided by either parent(s)/guardian, health care provider, school system, etc). Uniquely, the use of relatable avatars was lauded relative to the standard provision of sexual health education. Moreover, end users conveyed that MyPEEPS was useful for those with little to no sexual health education:

I think it’s also nice because it’s very educational and kind of starts easy, like you don’t have to have any prior knowledge, like any sexual education, before. The app really provides you with a lot of baseline sexual education, which is really important.

Chicago, 24 years

Acknowledging the individual variability in sexual experience, end users found the app to be useful for those who have less sexual experience or knowledge:

I thought it did a great job about...talking about the bottom line, like standing by the things that are important to you. I could see as someone who didn’t have much information about sexual education and maybe didn’t know what the risks of different sexual activities were they would get more information and that would change their bottom line.

Chicago, 24 years

### Effort Expectancy

Effort expectancy is defined as the degree of ease associated with the use of MyPEEPS, including *ease of use* and *complexity*. Most end users described the mobile app as *easy to use*. For example, one participant said:

I felt the flow of the app was really easy to follow and set up in a very nice way.

New York, 19 years

Most end users found the overall interface of MyPEEPS to be intuitive, approachable, and easy to use:

Well it’s pretty straightforward and easy to use. The logo is cute.

New York, 20 years

The MyPEEPS activity map was designed as an urban city street, reflecting participant’s environment. Overall, participants at these study sites related to the map design. Reflecting on the ease of navigation, one participant noted:

I loved the platform of scrolling through the street. I thought that was really cool because you can see what’s still to come. While they’re only brief little titles you can still see what’s going on, you can see there is going to be a point in which you’re going to see the experience of a clinic and things of that sort that I enjoyed. It was, of course, really easy to go back and click on other things if I did want to go back.

Chicago, 24 years

On the other hand, one user noted:

I didn’t like how it was so linear.

Chicago, 25 years

**Figure 2:**
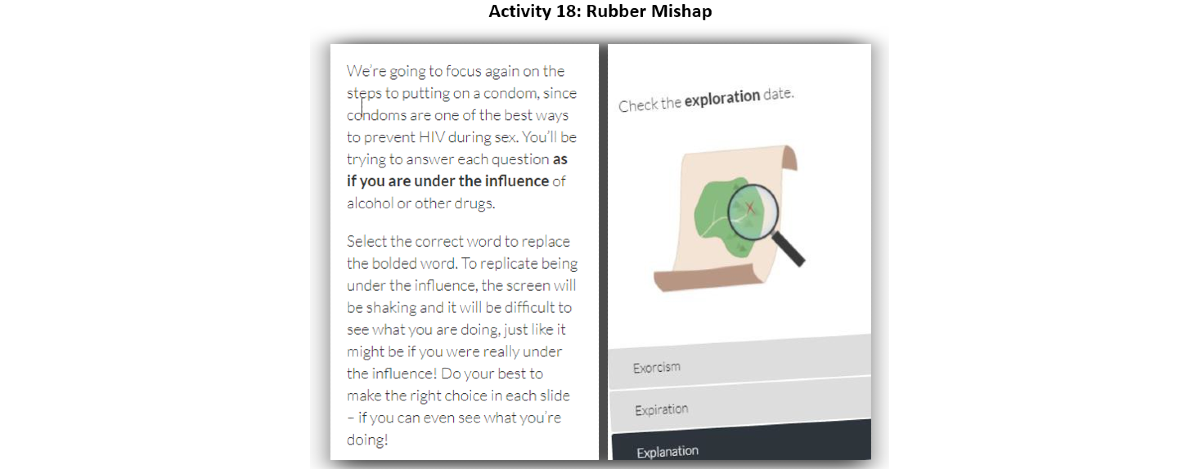
App activity with the screen shaking to simulate being under the influence of drugs or alcohol.

Furthermore, although there were very few negative comments about the gamification aspects of the app, most end users found activity 18 (Figure 2), an interactive feature designed to simulate the experience of intoxication, to be hard to use with the game aspect distracting from the educational purpose. For example, one end user commented:

With 18 the thing started shaking so I couldn’t read the thing properly. I think they should change that. If you ever have it shake, don’t have it shake too much.

New York, 19 years

### Social Influence

Social influence was applied in this study as the degree to which an individual perceives that important others believe that they would benefit from the use of MyPEEPS app [32]. Subconstructs included (1) *subjective norms* and (2) *social factors*.

*Subjective norms* are the end users’ perception that most people who are important to him think that he should or should not use the MyPEEPS app. Several end users detailed that they would share this mobile app with their peer(s) and/or current sexual partner(s):

Me and my lover have to plan. I know I will be looking at the app. When I get home, I am going to tell him all about it.

New York, 19 yeas

Conversely, other participants noted that they would not share this app or content with their partner(s) or friend(s) because of embarrassment, implied stigma, or discrimination:

I feel like for friends I would be okay telling them. If it’s like a partner I might be embarrassed. It seems a little bit like I don’t know what I am doing so I have to use this app to help support me make all my decisions. For friends I feel like I’d be more open to share that with them.

New York, 23 years

Social factors are the end user’s perspective of their subjective culture (ie, YMSM) and physical environment [32]. This construct was adapted to include emergent subthemes derived from the analyses that may influence the core determinants of usage intention and behavior, including privacy, age, gender, sexuality, and language.

#### Privacy

Participants’ reported use of the app was influenced by their physical and social environment and sense of privacy while completing app activities. Privacy was a salient concern because of the sensitivity of sexual health content and associated concern for being “outed”:

Also, the video was really blunt and direct, and so if I was not listening to that without headphones...it was like not safe for work kind of thing. And if my parents were around, I would feel super uncomfortable. Yeah. And just like making sure that I have privacy. I mean, this stuff is pretty blunt. And I mean, that’s good, but at the same time being aware of who’s around is kind of important.

New York, 23 years

#### Age

Most end users found the applicability of the content depending on age of the user. A representative quote is as follows:

Yes, and I think specifically younger adults would benefit from this. If I were a college student still in my freshman year, this would be perfect for some sort of training. I think it would be really, effective for young people who are going out into the world, like 18/19 years old...It gives you strategies. I think that a lot of young people probably don’t get the sex education they need until college, and not everyone goes to college.

Chicago, 25 years

Similarly, some participants commented that the scenarios are most relevant for those still living with parents or caregivers:

It wouldn’t really be as relevant if I'm in my mid-twenties...because you are in different environments. You may not be living with your parents...and you're navigating different other social circles, and networking in other communities. Some of the scenarios could be transferred over.

New York, 20 years

Another participant reflected:

I think maybe for the younger crowd; the visual style can maybe be updated, to be a little more grown-up.

New York, 21 years

Some end users commented about the app’s graphics being tailored to a younger audience, using descriptors such as “juvenile,” “cartoonish,” and “elementary.” One participant commented on the following for improving the interface for young adults aged 19 to 25 years:

It’s just tricky, because I appreciated the creativity there, but it did feel a little elementary.

Chicago, 24 years

#### Gender/Sexuality

Participants discussed the variability of gender expression among YMSM, which was reflected in the study sample, including participants who described themselves as nonbinary, two-spirit, masculine of center, and feminine of center. One participant noted:

Something I also appreciated about the app was it was representative of a lot of different identities that’s you could have, and it also talked about the intersection of identities, like gender identity and sexual orientation and race and how all those can play together and affect someone’s experience.

Chicago, 24 years

Thus, the match of key characteristics in the MyPEEPS Mobile app is likely to resonate with participants.

In addition, several participants noted the desire for a narrative or an avatar who was in the process of “coming out,” amid the stages of disclosing one’s sexuality and/or gender. For instance:

How would you address coming out to parents, or addressing to your friends that I think I might be into guys or girls or look, I have these feelings, like how to navigate those effective communicating strategies.

New York, 21 years

#### Language

Most participants related to language used in the delivery of content:

Very sex positive, for sure, using vocabulary that we use; And this is all really very accessibly worded...if that’s a word...simply worded, simply put.

New York, 23 years

In contrast, several participants did not relate to the use of vernacular language:

I felt like, at that moment, some of the vocabulary I thought was intrusive.

New York, 22 years

Overall, participants found the language used by avatars during quizzes and in case scenarios to be appropriate.

## Discussion

### Principal Findings

As mHealth interventions become increasingly available for consumers, it is critical to ensure that mobile technologies are designed and targeted to meet end users’ needs [34]. Given that this app was designed for YMSM aged 13 to 18 years, a rigorous evaluation of its usability in YMSM aged 19 to 25 years is important to understand whether it is an acceptable intervention for an older demographic age group.

Overall, end users of this study found the mobile app to be highly usable, as indicated through the survey data (Health-ITUES and PSSUQ), with no major bugs or functional problems reported and no issues with flow of activities. However, the qualitative analyses, based on the UTAUT framework [32,33], provided important insight into nuances of both the strengths and limitations of the app content, including the overall intervention approach, activities, and images for young adult MSM. Thus, emergent qualitative findings provided context and further allowed for in-depth evaluation from the user’s perspective.

The basic sexual health information in the app was deemed useful overall, with limitations for those with preexisting sexual health knowledge or sexual experience. The app was perceived to be most useful among those with limited sexual health knowledge and experience. To optimize content for a broader group of young adult MSM, more advanced educational content and social scenarios may need to be added.

Users reported the relative advantage of the MyPEEPS app over standard sexual health approaches because of the use of avatars and animation to aid in the understanding and absorption of content. The use of the avatars also provided a relative advantage, providing material that is salient and relatable to YMSM.

Regarding the UTAUT construct of effort expectancy, the app menu, which was depicted as an urban city street in which participants advanced along activities sequentially, moving horizontally along the street scene, was largely perceived as both easy to navigate and interesting. Although there were no functional problems with app activities, one activity in which the screen shakes to simulate intoxication was perceived as distracting, with the related text difficult to read, limiting its effectiveness.

Social influence encompassed subconstructs including social factors and subjective norms. Although many participants lauded the “sex positive” nature of the app, basic educational information, and frank language about sexual behavior, they found that these may result in discomfort on the part of the user to complete the activities in front of others because of the stigma associated with the content, reflecting the subjective norms of those around them. Although users are encouraged to use the app in private, this was reinforced given these comments.

The participants were universally enthusiastic about communicating and recognizing different gender presentations and identities in the app and the recognition of intersecting multiple identities, including gender, race/ethnicity, and sexual orientation. Participants recommended a case scenario of an avatar who is in the process of “coming out” disclosure of one’s gender and/or sexuality. Although “coming out” is a common thread in the community, the process of disclosing one’s sexual and gender identity is dependent on one’s circumstance and social context and intersecting social identities [9]. This process may be explained as YMSM nascent from adolescence into young adulthood; an emergence of self-concept and independence is developed through (1) identity formation—awareness and exploration of one’s sexual and gender identity—and (2) identity integration—involvement in, comfort with, and disclosure of one’s sexual and/or gender identity [42]. As these processes are tangential and nonlinear, the majority of participants responded that MyPEEPS is a foreseeably useful tool for MSM who do not have access to or are impeded by other contextual social barriers to comprehensive sexual health education.

Although there are privacy and security concerns with the use of mobile technology for storing personal health information [43], educational mobile apps such as MyPEEPS may be useful for protecting privacy for highly stigmatized topics such as sexual health for MSM, HIV/AIDS education, gender identity, and mental health [21,23,44]. In fact, MyPEEPS can be very useful for YMSM who are fearful of disclosing their sexuality to family or providers and want to access information about how to protect their health. Thus, MyPEEPS may be particularly useful to those students who do not want to disclose their sexual or gender identities or to those who are living in regional areas with less access to comprehensive sexual health education (ie, varying abstinence policies at the state and school district level) [45].

### Limitations

Our study presents some limitations. First, the purposive sample of young adult MSM from urban settings may not generalize to suburban or rural settings and individuals therein. In addition, this study did not include transgender men (assigned female at birth) who have sex with men, and therefore, findings cannot be generalized to that group.

### Conclusions

Critical to the uptake and use of eHealth interventions is the rigor in which they are appraised before implementation [26,46,47]. Taking these factors into consideration, this study aimed to rigorously appraise the potential use of the MyPEEPS Mobile intervention, designed for adolescent MSM, to young adult MSM, aged 19 to 25 years, with the goal of future application and/or adaptation to this older group. The perception of usability and acceptability of MyPEEPS Mobile among this demographic of young adult MSM was overall favorable but with key recommendations to improve the applicability of the intervention material for this group with more sexual education and sexual experience.

## Appendix

Multimedia Appendix 1 Screenshots from the Male Youth Pursuing Empowerment, Education and Prevention Around Sexuality Activities app.

## Abbreviations

Health-ITUES: Health Information Technology Usability Evaluation Scale
IRB: Institutional Review Board
mHealth: mobile health
MSM: men who have sex with men
MyPEEPS: Male Youth Pursuing Empowerment, Education and Prevention around Sexuality
PrEP: pre-exposure prophylaxis
PSSUQ: Post-Study System Usability Questionnaire
STI: sexually transmitted infection
UTAUT: Unified Theory of Acceptance and Use of Technology
YMSM: young men who have sex with men
