# Supplementary material for: A Mobile Sexual Health App on Empowerment, Education, and Prevention for Young Adult Men (MyPEEPS Mobile): Acceptability and Usability Evaluation
Source: JMIR Form Res. 2020 Apr 7;4(4):e17901. doi: 10.2196/17901 (PMC7175191; doi:10.2196/17901)
Supplement: Multimedia Appendix 1 [file formative_v4i4e17901_app1.docx]

**Activity 2: Bottomline**

**Activity 3: Underwear Personality Quiz**

**Activity 1: Profile Set Up**

**Activity 5: P’s On-Again Off-Again Bottomline**

**Activity 4: My Bulls-I**

**Activity 6: Sexy Settings**

**Activity 9: HIV True/False**

**Activity 8: Step Up, Step Back**

**Activity 7: Goin’ Downhill Fast**

**Activity 10: Checking In On Your Bottomline**

**Activity 12: Testing with Tommy**

**Activity 11: P Gets Woke About Safer Sex**

**Activity 15: Checking In On Your Bottomline Again**

**Activity 14: Ordering Steps to Effective Condom Use**

**Activity 13: Well Hung**

**Activity 18: Rubber Mishap**

**Activity 17: 4 Ways to Manage Stigma**

**Activity 16: Peep In Love**

**Activity 21: Bottomline Overview**

**Activity 20: Last Time Checking In On Your Bottomline**

**Activity 19: Get a Clue!**
